# Wavelength Selection for NIR Spectroscopy Based on the Binary Dragonfly Algorithm

**DOI:** 10.3390/molecules24030421

**Published:** 2019-01-24

**Authors:** Yuanyuan Chen, Zhibin Wang

**Affiliations:** 1 School of Information and Communication Engineering, North University of China, Taiyuan 030051, China; 2 Engineering Technology Research Center of Shanxi Province for Opto-Electronic Information and Instrument, North University of China, Taiyuan 030051, China; wangzhibin@nuc.edu.cn; 3 School of Science, North University of China, Taiyuan 030051, China

**Keywords:** wavelength selection, NIR spectroscopy, binary dragonfly algorithm, ensemble learning, quantitative analysis modeling

## Abstract

Wavelength selection is an important preprocessing issue in near-infrared (NIR) spectroscopy analysis and modeling. Swarm optimization algorithms (such as genetic algorithm, bat algorithm, etc.) have been successfully applied to select the most effective wavelengths in previous studies. However, these algorithms suffer from the problem of unrobustness, which means that the selected wavelengths of each optimization are different. To solve this problem, this paper proposes a novel wavelength selection method based on the binary dragonfly algorithm (BDA), which includes three typical frameworks: single-BDA, multi-BDA, ensemble learning-based BDA settings. The experimental results for the public gasoline NIR spectroscopy dataset showed that: (1) By using the multi-BDA and ensemble learning-based BDA methods, the stability of wavelength selection can improve; (2) With respect to the generalized performance of the quantitative analysis model, the model established with the wavelengths selected by using the multi-BDA and the ensemble learning-based BDA methods outperformed the single-BDA method. The results also indicated that the proposed method is not limited to the dragonfly algorithm but can also be combined with other swarm optimization algorithms. In addition, the ensemble learning idea can be applied to other feature selection areas to obtain more robust results.

## 1. Introduction

Over the past decades, near-infrared (NIR) spectroscopy has been successfully applied in many areas, such as agriculture, medicine, environment [1]. Compared with traditional laboratory methods, NIR has the advantages of rapid speed and noninvasiveness. Usually, the NIR spectra collected from samples have the following several characteristics. Firstly, the number of necessary wavelengths is far higher than the number of samples, which, from the point of view of multivariate equations, means that the number of X factors (wavelengths) is far higher than the number of equations (samples). Hence, it is impossible to obtain a unique solution. Secondly, the contribution of each wavelength to quantitative analysis is different, since some wavelengths may be strongly correlated with the target content, while others may show little or no correlation. Obviously, it is not possible to consider the whole range of NIR spectra to perform subsequent qualitive or quantitative analysis. Hence, wavelength selection (also called “feature selection” and “variable selection”) is an essential preprocessing step to find the most representative wavelengths and eliminate uninformative wavelengths.

In recent years, many researchers have focused on the wavelength selection issue and proposed a series of algorithms which have been proven effective in many areas. For example, Norgaard et al. [2] proposed the interval PLS (iPLS) method, which first divides the whole range of spectrum into several intervals and then adopts a forward/backward stepwise selection algorithm to choose the most effective interval combinations. However, in how many intervals should the whole range of the spectrum be divided? Should it be divided equally or non-equally? These factors have a great influence on the wavelength selection results. Centner et al. [3] proposed a novel feature selection method called uninformative variable elimination (UVE), which brings in some random variables as the criterion of evaluating the correlation between wavelength and output target component. If the correlation coefficient of a certain wavelength is smaller than the random variables, the wavelength variable can be eliminated as an uninformative variable. The idea of the UVE algorithm is ingenious and intuitionistic. However, the disadvantage of this method is that the number of selected wavelengths is commonly large because this approach can only eliminate the uninformative wavelengths without selecting the most representative wavelengths [4]. Our group previously proposed an L1 regularization-based wavelength selection method [5], which considered the wavelength selection problem as a sparsity optimization issue. By adjusting the value of the sparsity parameter λ, we can freely control the number of selected wavelengths, which is reduced while the value of λ increases. Detailed information can be found in reference [5].

Besides the above-mentioned methods, evolution and swarm optimization methods (such as genetic algorithm [6], bat algorithm [7], particle swarm optimization [1], etc.) have also been applied to solve the wavelength selection problem. The main idea at the basis of these methods is similar. Firstly, they imply the generation of an initial population which is comprised of some individuals, each of which is a binary sequence. The length of each binary sequence is equal to the number of wavelengths, and the value of each point in the binary sequence is “1” or “0”, indicating if the corresponding wavelength is selected or not. Secondly, the iterative searching is implemented by a series of heuristic strategies (for example, selection, crossover, mutation operators in genetic algorithm). However, due to the fact that there are some random mechanisms in swarm optimization methods (such as roulette wheel selection, Lévy flight, etc.), the wavelength selection results of each optimization are variable; hence, it is often confusing which wavelength variables should be taken into account. In 2016, Mirjalili et al. [8,9] proposed a novel swarm optimization method called “dragonfly algorithm (DA)” based on the observation, summary, and abstraction of the behaviors of the dragonfly in nature. Hence, the main contribution of this paper is to apply the binary dragonfly algorithm to solve the wavelength selection problem and improve its stability on the basis of the ensemble learning method.

The paper is organized as follows. Section 2 introduces the principles of the dragonfly algorithm and the basic idea of the transition dragonfly algorithm from continuous domain to binary domain. The proposed algorithm is introduced in detail in Section 3. The experimental results and discussion are presented in Section 4. Finally, Section 5 summarizes the contribution of this work and suggests some directions for future studies.

## 2. Dragonfly Algorithm

In the following section, we will introduce the principles of the dragonfly algorithm in continuous and discrete domains.

### 2.1. Continuous Dragonfly Algorithm

Biologists found that dragonflies have two interesting swarming behaviors: static and dynamic. This observation inspired the design of the dragonfly algorithm because there are two similar phases (called exploration and exploitation) in traditional swarm optimization methods. In the static swarm mode, dragonflies fly over different directions in a small area, which corresponds to the exploration phase; in the dynamic swarm mode, dragonflies fly in a bigger area along one direction, which is the main objective of the exploitation phase.

**Figure 1:**
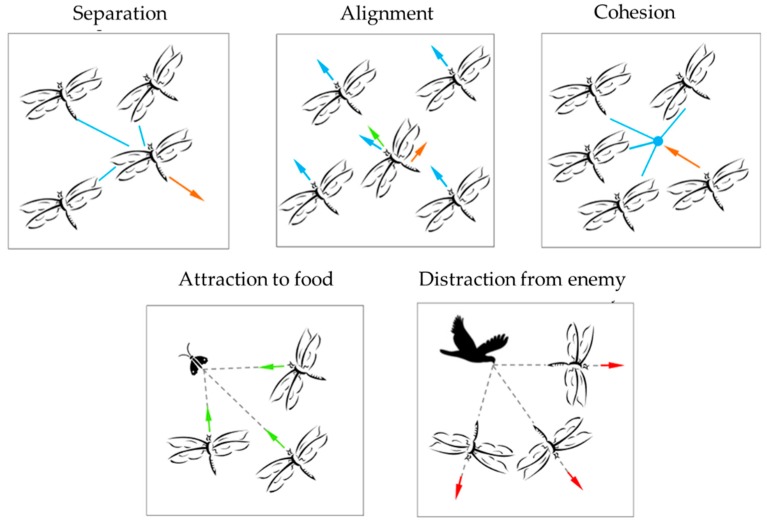
Five main strategies by which dragonflies change their position [8].

The main task of dragonflies is trying their best to survive; hence, they should be attracted towards food sources while avoiding enemies. Biologists observed that dragonflies usually change their position through five main strategies: separation, alignment, cohesion, attraction to food, and distraction from enemy, as shown in Figure 1.

The strategies to change position are mathematically modeled, as shown in Table 1.

As seen from the above table, dragonflies tend to align their flying while maintaining proper separation and cohesion in the dynamic swarm manner. However, while in the static swarm manner, alignments are very low, and cohesion is high to attack preys. Hence, we can assign high alignment and low cohesion weights to dragonflies exploring the search space, and low alignment and high cohesion to dragonflies exploiting the search space. In the transition between exploration and exploitation, the radius of the neighborhood is increased proportionally to the number of iterations. Another way to balance exploration and exploitation is to adaptively tune the swarming factors (s, a, c, f, e, and w) during the optimization. Additionally, to improve the randomness, stochastic behavior, and exploration of artificial dragonflies, dragonflies are required to fly around the search space using a random walk (*Lévy* flight) when there are no neighboring solutions. More detailed information can be found in reference [8].

### 2.2. Binary Dragonfly Algorithm (BDA)

In the continuous domain, dragonflies are able to update their positions through adding the step vector ∆X to the position vector X. However, in the discrete domain, the position of dragonflies cannot be updated in this way, because the position vectors X can only be 0 or 1. There are many transition methods described in the literature mapping the transition from continuous domain to discrete domain. Among them, the easiest and most effective method is to employ a transfer function which receives velocity values as inputs and returns a number in [0, 1], representing the probability of the changing positions.

There are two types of transfer functions: s-shaped and v-shaped. According to Saremi et al. [10], the v-shaped transfer functions outperform the s-shaped transfer functions because they do not force particles to take values of 0 or 1. In this paper, the following transfer function is utilized [11]:

(1) $$T\left(∆x\right)=\left|\frac{∆x}{\sqrt{∆x^{2}+1}}\right|$$

After calculating the probability of changing position for all dragonflies, the following position-updating formula is employed to update the position of dragonflies in binary search spaces:

(2) $$X_{t+1}=\{\begin{array}{l} -X_{t},\;r<T\left(∆x_{t+1}\right)\\ X_{t},\;r\geq T\left(∆x_{t+1}\right)\; \end{array}$$

where r is a number in [0, 1].

## 3. Wavelength Selection Framework Based on BDA and Ensemble Learning

**Figure 2:**
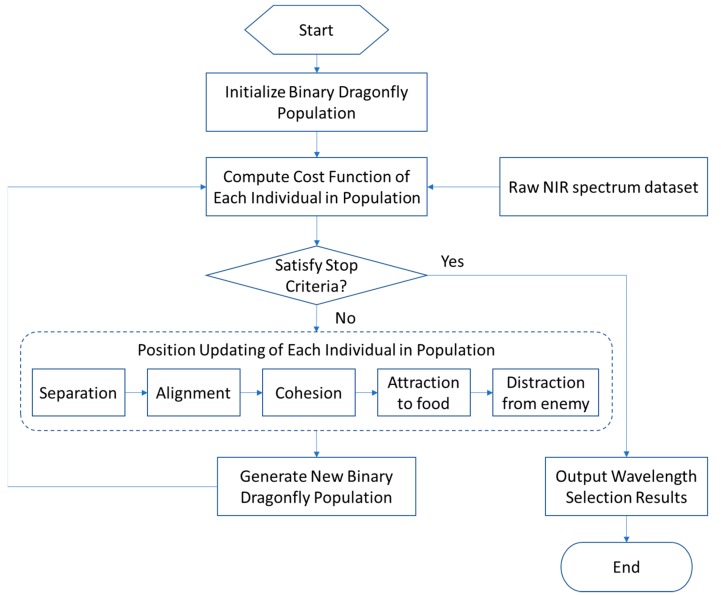
The flowchart of the binary dragonfly algorithm (BDA)-based wavelength selection method.

The flowchart of BDA-based wavelength selection method is illustrated in Figure 2. The main procedures are as follows.

**Step 1:** Mapping the wavelength selection problem to the BDA optimization problem. More precisely, initialize the binary dragonfly population. Without loss of generality, suppose there are K wavelengths in the whole range, hence the individuals in the initialized population are in binary series whose length is equal to K, in which the values of “1” and “0” indicate if the corresponding wavelength is selected or not.

**Step 2:** Evaluating the “goodness” or “badness” of each individual in the initialized population. For each individual, find the selected wavelength combinations and then establish the quantitative analysis model to predict the content of octane by using the partial least-squares (PLS) method. In this paper, the cost function (similar to the fitness function in the genetic algorithm) is defined as the root-mean-square error of cross validation (RMSECV) of the quantitative analysis model, which means that the individual with the smallest RMSECV has a good wavelength combination.

**Step 3:** Judging whether the stop criteria are satisfied or not. If yes, output the best individual in the population as the selected wavelength. If no, go to **step 4**. Generally, there are two approaches to design the stop criteria. The first takes the maximum iterations as the stop criteria, while the second takes the absolute error between two successive iterations as the stop criteria.

**Step 4:** According to Table 1, each individual in the population updates its position through five strategies, including separation, alignment, cohesion, attraction to food, and distraction from enemy. Finally, a new binary dragonfly population will be generated.

**Step 5:** Go back to **Step 2**, calculate the cost function of each individual in the new population, then execute the loop until the stop criteria are satisfied.

**Figure 3:**
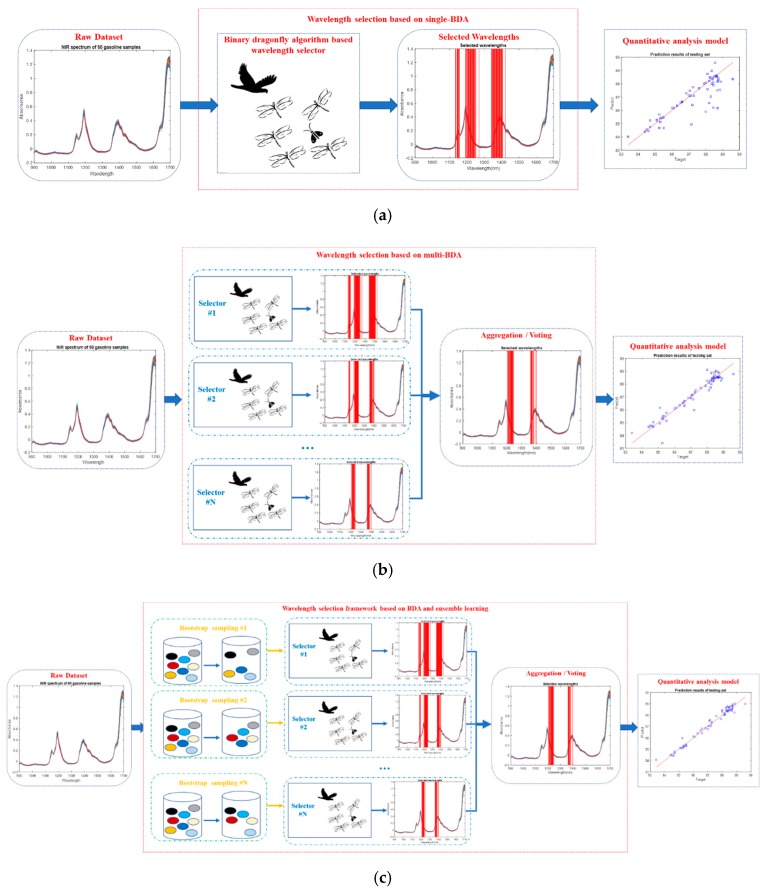
Three typical wavelength selection methods based on BDA. (**a**) Wavelength selection based on the single-BDA method; (**b**) wavelength selection based on the multi-BDA method; (**c**) wavelength selection framework based on the BDA and ensemble learning method.

In this paper, we propose three typical wavelength selection methods based on BDA, as illustrated in Figure 3.

Similar to traditional swarm optimization methods, the wavelength section algorithm was designed on the basis of the single BDA. Figure 3a shows the procedure, which consists in using the BDA only once to search the most representative wavelengths. As mentioned above, because there are some random mechanisms in BDA, the wavelength selection results of each search are different.

To solve this problem, Figure 3b illustrates a possible solution which was designed on the basis of the multi-BDA. Differently from the single-BDA method, in this case the BDA is used many times, and then all the resulting selected wavelengths are aggregated by a voting strategy; those wavelengths with high votes are selected as the final wavelengths. However, an issue needs to be considered, that is, what is the quantitative criterion of “high votes”? Should it be 60%, 70%, 80%, or higher? The higher the vote percentage, the lower the number of selected wavelengths. If the number of selected wavelengths is too small, the performance of the subsequent quantitative analysis model may be reduced. Therefore, reaching a tradeoff between them is a challenge.

Furthermore, we propose a novel wavelength selection framework based on BDA and ensemble learning, as shown in Figure 3c. Traditionally, ensemble learning is often used to combine several models to improve the generalized performance of a model. However, in this paper, ensemble learning was not used to improve the model’s performance. Instead, the idea of traditional ensemble learning was introduced to improve the stability of the wavelength selection results. Hence, only one PLS model was used during the wavelength selection period. Compared with the multi-BDA method, before the BDA searching procedure, Bootstrap sampling is added to randomly generate a series of different subsets. Suppose there are N samples in the raw dataset: N samples are drawn from the raw dataset with replacement, and then replicated samples are eliminated; hence, usually, the size of the subset becomes smaller because of the elimination.

Similar to the traditional swarm optimization algorithms, the single-BDA method also suffers from instability. In contrast, the multi-BDA and ensemble learning-based BDA methods can improve the stability. The key technical skill is to reduce the randomness inherent in the BDA. Even if both multi-BDA and ensemble learning-based BDA can achieve this, their principles are different. Although the multi-BDA method has many BDA selectors, they are built on the whole raw dataset. In contrast, the BDA selectors in the ensemble leaning method are built on different subsets. The variety of subsets leads to the different wavelength selection results from each BDA. This is the essence of ensemble learning. Besides, the computational complexity of the ensemble learning-based BDA method is smaller than that of the multi-BDA method, which mainly reflects in the computation of the cost (fitness) function RMSECV.

First of all, let us look at the scenario of the multi-BDA method. For example, consider a raw dataset with 100 samples and suppose the raw dataset is uniformly divided into five folds, each of which contains 20 samples. Establish five PLS models, each of which is trained on the basis of four folds (80) samples and tested on the remaining (20) samples.

Next, let us look at the scenario of the ensemble learning-based BDA method. According to the theoretical analysis, the size of subsets generated by Bootstrap sampling is approximately 63.2% that of the raw dataset (the whole theoretical analysis process can be found in reference [12]). Hence, there are about 63 samples in the subsets, and, similar to the multi-BDA method, the subset is uniformly divided into five folds, each of which contains about 12 samples. Establish five PLS models, each of which is trained on the basis of four folds (about 48) samples and tested on the remaining (12) samples.

The experimental results proved that, though the size of the samples in the subset became smaller, the performance was close to that of the multi-BDA method.

## 4. Experimental Results and Discussion

To validate the performance of the proposed wavelength selection methods, we applied the above-mentioned three typical algorithms to the public gasoline NIR spectroscopy dataset. The main aim was to find the most representative wavelengths to predict the content of octane by NIR spectrometry on the basis of the binary dragonfly algorithm.

### 4.1. Dataset Description

**Figure 4:**
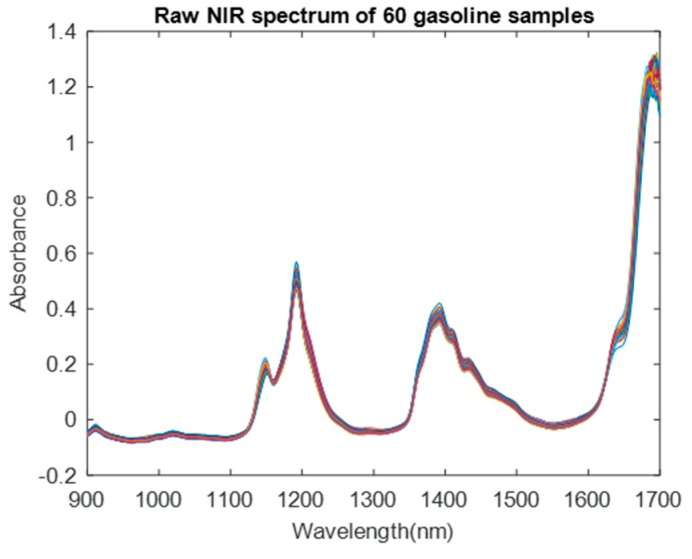
Raw spectrum of 60 gasoline samples.

This dataset consists of 60 samples of gasoline measured by NIR spectrometry. The wavelength range was 900–1700 nm at 2 nm intervals (401 channels). The octane value for each of the samples was also included [13]. The raw whole range of the NIR spectrum of 60 samples is illustrated in Figure 4. Because this dataset had been embedded into MATLAB software (MathWorks Inc., Natick, MA, USA), the results were implemented in MATLAB R2017b. The basic source code of the BDA could be downloaded from the inventor’s personal website (http://www.alimirjalili.com/DA.html) or MathWorks official website (https://www.mathworks.com/matlabcentral/fileexchange/51032-bda-binary-dragonfly-algorithm). On the basis of this source code, we implemented the single-BDA, multi-BDA, and ensemble learning-based wavelength selection codes.

### 4.2. Experimental Results

Firstly, the single-BDA method was applied to select the most representative wavelengths. In this paper, the PLS method was adopted to establish a quantitative analysis model. With respect to each binary individual, firstly, those wavelengths with value “1” were selected, and then a quantitative analysis model was established on the basis of these wavelengths. The values of parameters in Table 1 were adaptively tuned according to references [8,9,10]; the values of the remaining parameters of the BDA are listed in Table 2; we determined them by trial and error. The influence of different parameter values on the performance of the quantitative analysis model will be introduced in detail in the discussion section. Additionally, the BDA was repeated 10 times in the multi-BDA method; also, 10 parallel BDA wavelength selectors were present in the ensemble learning method. The influence of the BDA repetition on the wavelength selection results will also be introduced in the discussion section.

**Figure 5:**
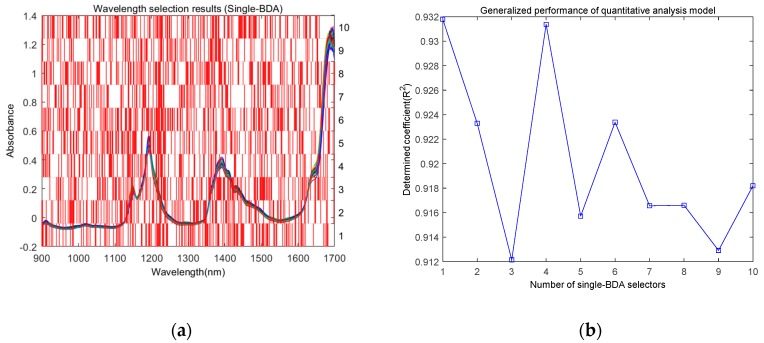
Wavelength selection results using the single-BDA method. (**a**) Wavelength selection results by applying a 10 times search; (**b**) corresponding generalized performance of the quantitative analysis model.

As mentioned above, because of the random mechanism in the BDA, the wavelength selection results of each search were different. Figure 5 illustrates the wavelength selection results by using the single-BDA method. We implemented it for 10 times, and the wavelength selection results of each search are shown in Figure 5a, from which it is evident that the results were different. The corresponding generalized performances of the quantitative analysis model established with these selected wavelengths were also different, and the determined coefficient R^2^ varied from 0.912 to 0.932, as shown in Figure 5b. These results indicated that, similar to traditional swarm optimization methods, the BDA can also be applied to solve the wavelength selection problem. However, it is often confusing which wavelength variables should be selected.

**Figure 6:**
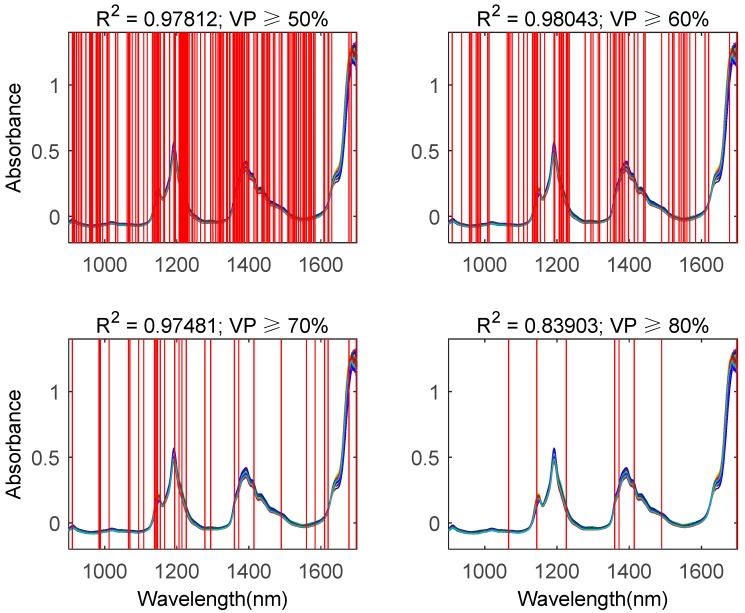
Wavelength selection results by using the multi-BDA method. VP: votes percentage. Upper left, upper right, lower left, and lower right represent selected wavelengths while the votes percentage is equal to or greater than 50%, 60%, 70%, and 80%, respectively.

As mentioned above, the difference between the multi-BDA and the single- BDA methods is that there as a voting strategy to aggregate the wavelength selection results of the multiple-time search. The experimental results of the multi-BDA method are shown in Figure 6, from which it is obvious that by adjusting the value of the votes percentage (VP), we can control the number of selected wavelengths. Actually, if VP is equal to or smaller than 40%, the number of selected wavelengths is so large that the dimension is still high; on the contrary, if the VP is equal to or greater than 80%, there might be no wavelength satisfying this criterion. Hence, here we only show the wavelength selection results with VP between 50% and 80%. By comparing Figure 6 to Figure 5b, we can see that the determined coefficient R^2^ of the quantitative analysis model established on the basis of the wavelengths selected with the multi-BDA method outperformed that obtained with the single-BDA method. However, the case of VP ≥ 80% was an exception, as only 7 wavelengths were selected. This means that if the number of selected wavelengths is too small, the generalized performance of the quantitative analysis model may decrease. Hence, a tradeoff has to be reached.

**Figure 7:**
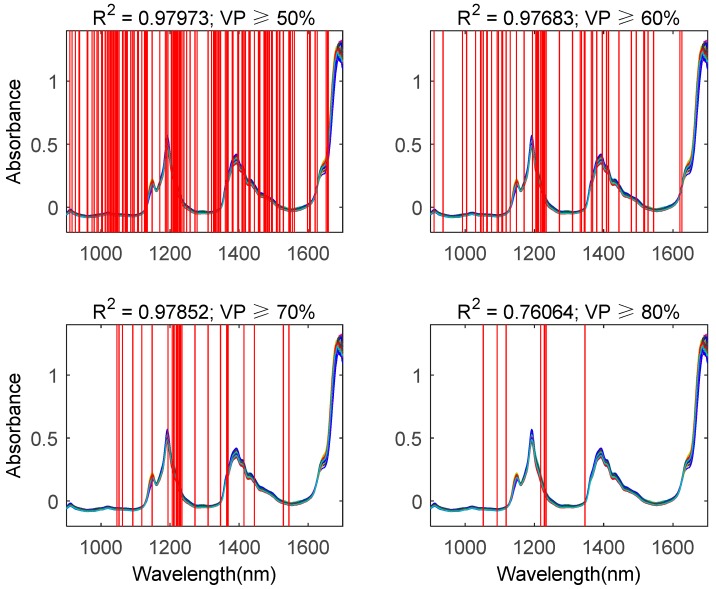
Wavelength selection results by using the BDA and ensemble learning method. VP: votes percentage. Upper left, upper right, lower left, and lower right represent selected wavelengths with votes percentage equal to or greater than 50%, 60%, 70%, and 80%, respectively.

Figure 7 describes the wavelength selection results by using the BDA and the ensemble learning method. The difference between this method and the multi-BDA method is that a series of Bootstrap sampling generators were added before the BDA. Because Bootstrap sampling is a method of random sampling with replacement, the size of the subset is smaller than the whole dataset. As mentioned above, the subset only contained about 63.2% samples of the whole set. By comparing Figure 7 to Figure 6, it is easy to see that, although the sample size was reduced through Bootstrap sampling, the generalized performance of the quantitative analysis models with selected features was close to that of the multi-BDA method. Hence, by using this method, we could reduce the computational complexity. However, similar to the multi-BDA method, if the votes percentage is equal to or greater than 80%, the generalized performance of the quantitative analysis model decreases. In summary, we suggest that the votes percentage should be set between 60% and 70%.

### 4.3. Discussion

#### 4.3.1. Influence of the Values of the BDA Parameters on the Generalized Performance of the Model

Similar to other evolution and swarm optimization algorithms, there are some parameters (as listed in Table 1 and Table 2) inherent in BDA and while the values of these parameters are different, the corresponding wavelength selection results will different too. Hence, we want to quantitatively analyze the influence of the values of these parameters on the wavelength selection results.

As mentioned above, in this paper the values of parameters in Table 1 were adaptively tuned as follows:

(3) $$w=0.9-iter\times \frac{0.9-0.4}{\max\_iter}$$

where iter and max_iter is the current and maximum iteration, respectively. It is obviously to see that w is linearly decreased from 0.9 to 0.4. 

With respect to parameter s, a and c, the adaptive tuning formula is:

(4) x=2×γ×θ, x∈{s, a, c}

where γ is a random between 0 and 1.

(5) $$\theta =\{\begin{array}{l} 0.1-iter\times \frac{0.1-0}{\max\_iter/2},\;iter\;\leq max\_iter/2\\ 0,\;iter\;>\;max\_iter/2 \end{array}$$

From Equations (4) and (5), we can find that the range of parameter s, a and c is between 0 and 0.2.

Parameter f is a random between 0 and 2, and parameter e is equal to θ.

**Figure 8:**
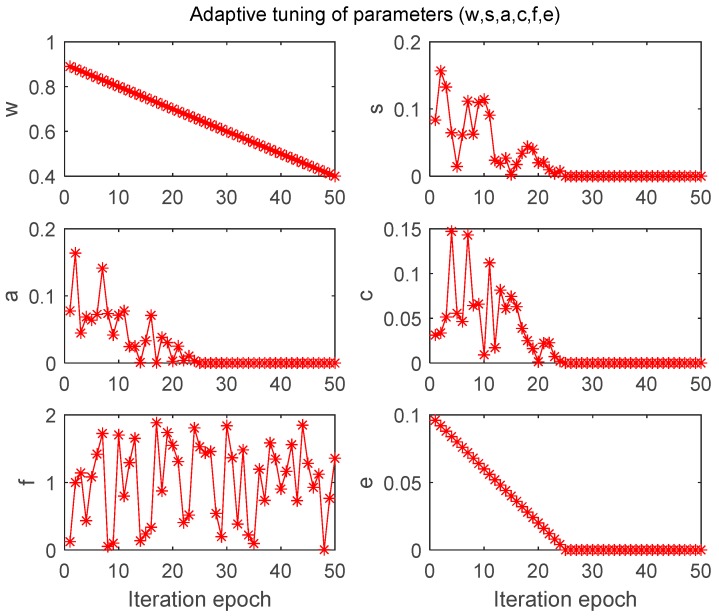
Adaptive tuning of parameters (w, s, a, c, f, e).

The adaptive tuning procedure of parameters (w, s, a, c, f, e) is illustrated in Figure 8. We can easily find that while the current iteration period is larger than half of maximum iteration, values of parameters s, a, c and e become zero. In other words, the separation, alignment, cohesion and detraction from enemy strategies were not included in the position updating procedure, and only inertia weight w and attraction to food f were considered.

Table 3 shows the influence of different parameter values on the generalized performance (RMSECV) of quantitative analysis model. The second column means that all of the parameters were adaptively tuned, and the third and fourth columns mean that the corresponding parameter’s value were fixed to its maximum or minimum respectively while other parameters were adaptively tuned. We can find that there is a trend that while these parameters were not adaptively tuned, the generalized performance (here we use mean RMSECV of 10 times repeated) of quantitative analysis model will decrease, which is consisting with reference [8].

**Figure 9:**
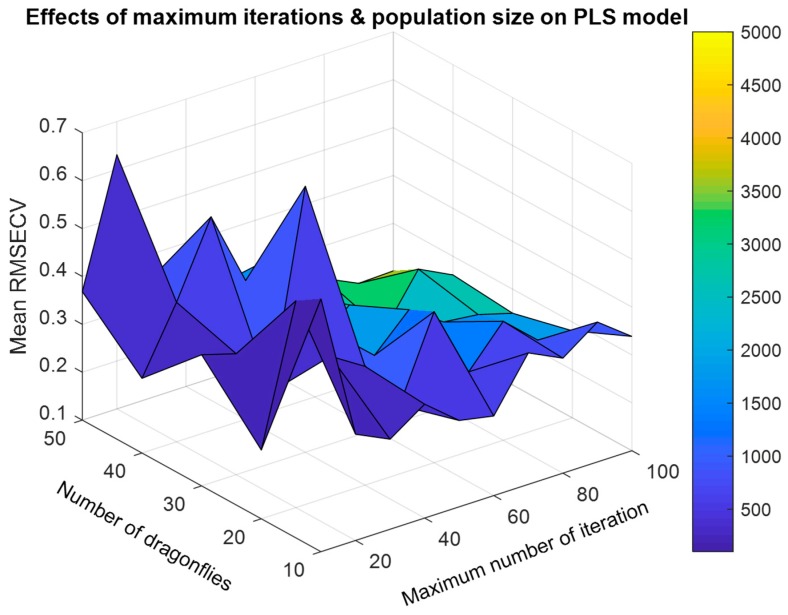
Influence of maximum number of iterations and number of dragonflies on the generalized performance of quantitative analysis model.

Further, we consider the parameters in Table 2. Previously principal component analysis (PCA) showed that the cumulative contribution of the first two principal components was higher than 90%, hence in this paper we take the first two principal components to establish the PLS regression model. Additionally, 5-fold cross validation is often used so that we can ignore its influence on the model’s performance. Hence, here we put focus on two parameters: maximum number of iterations and number of dragonflies. We implemented a two-dimensional grid to evaluate the influence of these two parameters, in which the maximum number of iterations and number of dragonflies were range from 10 to 100 and 10 to 50, respectively. Because of the limitation of space, here we only take single-BDA as an example. At each parameter value pairs, mean RMSECV of 10 times repeated PLS models with selected wavelengths was computed as the evaluation index. The experimental results were illustrated in Figure 9, from which we can obviously find that with the increase of maximum number of iteration and number of dragonflies (population size), mean RMSECV of 10 times repeated PLS models showed a decrease trend. while the computational complexity increases a lot. Hence, there is a trade-off between them.

#### 4.3.2. Comparison Between Proposed Methods and Traditional Methods

In order to validate the efficiency of proposed methods, here we compared the proposed single-BDA, multi-BDA and ensemble learning based BDA methods with traditional methods. Considering the fact that it is difficult to find a common standard to implement the comparison between proposed methods with interval PLS, hence here we limit the comparison between proposed methods with evolution and swarm optimization methods. Because our previous studies have validated the efficiency of binary bat algorithm and genetic algorithm, here we implemented the comparison between proposed BDA and binary bat algorithm and genetic algorithm. We set the population size and maximum of iteration of these methods were all same. As mentioned above, for each method, mean RMSECV of 10 times repeated PLS models with selected wavelengths was computed as the evaluation index. The comparison results were listed in Table 4, from which we can obviously find that compared with genetic algorithm and binary bat algorithm, the performance of single-BDA method is a little lower. However, there is no significant statistical difference between them. Additionally, the performance of multi-BDA and ensemble learning based BDA methods were similar, both outperforms traditional methods and single-BDA. 

## 5. Conclusions

In terms of the wavelength selection problem in NIR spectroscopy, this paper proposes a novel method based on binary dragonfly algorithms, which includes three typical frameworks: single-BDA, multi-BDA, and ensemble learning-based BDA framework. The experimental results for a public gasoline NIR dataset showed that by using the proposed method, we could improve the stability of the wavelength selection results through the multi-BDA and ensemble learning-based BDA methods. With respect to the subsequent quantitative analysis modeling, the wavelengths selected with the multi-BDA and ensemble learning BDA methods outperformed those selected with the single-BDA method. When comparing the ensemble learning-based BDA and the multi-BDA methods, it can be seen that they can provide similar wavelength selection results with lower computational complexity. The results also indicated that the proposed method is not limited to the dragonfly algorithm but it can be combined with other swarm optimization algorithms. In addition, the ensemble learning idea can be applied to other feature selection areas to obtain more robust results.

Besides, during the experimental results analysis, we found that, for the same selected wavelengths, there were great changes of the generalized performance (RMSECV) between different subsets. This means that not only the wavelength variables, but also the samples influence the generalized performance of the quantitative analysis model. Currently, the majority of studies are focused on the wavelength selection problem; we suggest that sample selection using swarm optimization methods is an open problem that needs to be studied further.

Additionally, in this study, we found that the votes percentage should be set between 60% and 70%. However, we do not know whether this range is also suitable for other datasets. This should be validated further. Actually, in general, we should not be concerned by which wavelengths are selected. Instead, we may want to know how good the quantitative analysis model will be by using the selected wavelengths. Hence, improving the generalized performance of the quantitative analysis model is a hot topic in NIR spectroscopy. With the development of artificial intelligence, ensemble learning methods (such as random forest, adaboost, etc.) and deep learning (such as convolutional neural networks [14,15,16,17,18,19]) will be mainstream in the future.

## Figures and Tables

**Table 1:** Mathematical modeling of the five main position-changing strategies of dragonfly [8].

Position Updating Strategies	Equations	Description
Separation	$S_{i}=-\sum_{j=1}^{N}X-\;X_{j}$	X: position of the current individual $X_{j}$: position of the *j*-th neighboring individual N: number of the neighboring individuals $V_{j}$: velocity of the *j*-th neighboring individual $X^{+}$: position of the food source $X^{-}$: position of the enemy (s, a, c, f, e): separation, alignment, cohesion, food, and enemy factors w: inertia weight t: current iteration
Alignment	$A_{i}=\frac{\sum_{j=1}^{N}V_{j}}{N}$	
Cohesion	$C_{i}=\frac{\sum_{j=1}^{N}X_{j}}{N}-X$	
Attraction to food	$F_{i}=X^{+}-X$	
Distraction from enemy	$E_{i}=X^{-}+X$	
Position updating	$∆X_{t+1}=\left(sS_{i}+aA_{i}+cC_{i}+fF_{i}+eE_{i}\right)+w∆X_{t}$ $X_{t+1}=\;X_{t}+\;∆X_{t+1}$	
Position updating with *Lévy* flight	$X_{t+1}=\;X_{t}+\;Levy\left(d\right)\times X_{t}$ *Lévy* $\left(x\right)=0.10\times \frac{r_{1}\times \sigma }{{\left|r_{2}\right|}^{\frac{1}{\beta }}}$ $\sigma =\;{\left(\frac{\Gamma \left(1+\beta \right)\times sin\left(\frac{\pi \beta }{2}\right)}{\Gamma \left(\frac{1+\beta }{2}\right)\times \beta \times 2^{\left(\frac{\beta -1}{2}\right)}}\right)}^{\frac{1}{\beta }}$ Γ(x)= (x−1)!	d: dimension of the position vectors $r_{1}$, $r_{2}$: random numbers in [0, 1] β: constant

**Table 2:** Values of the BDA parameters.

Parameters	Values
Maximum number of iterations	50
Number of dragonflies	10
Number of wavelengths	401
Separation, alignment, cohesion, food, and enemy factors	adaptive tuning
Number of principal components	2
Number of folds of cross validation	5

**Table 3:** The values of related parameters of BDA.

Parameters	Mean RMSECV of 10 Times Repeated PLS Models with Selected Wavelengths		
	Adaptive Tuning	Fixed (Maximum)	Fixed (Minimum)
w (0.4–0.9)	0.3801	0.5196	0.2583
s (0–0.2)		0.5500	0.3700
a (0–0.2)		0.4833	0.4186
c (0–0.2)		0.4955	0.4071
f (0–2)		0.4871	0.5224
e (0–0.1)		0.3796	0.4570

**Table 4:** Comparison between proposed BDA based methods and traditional methods.

Methods	Population Size	Maximum of Iteration	RMSECV of 10 Times Repeated PLS Models with Selected Wavelengths	
			Mean	Std
Genetic algorithm	20	50	0.4016	0.0624
Binary bat algorithm			0.3672	0.0482
Single-BDA			0.3801	0.0549
Multi-BDA			0.3265	0.0215
Ensemble learning based BDA			0.3294	0.0168
